# Supplementary material for: Interventions to Expand Community Pharmacists’ Scope of Practice
Source: Pharmacy (Basel). 2024 Jun 19;12(3):95. doi: 10.3390/pharmacy12030095 (PMC11207271; doi:10.3390/pharmacy12030095)

SUPPLEMENTARY TABLE S1: Search strategy [Search conducted on: 6 April 2023. Updated search conducted on: 13 May 2024]

Database	Search strategy
**Medline (ALL)**	1. exp Pharmacy/<br>2. Pharmacies/<br>3. Pharmacists/<br>4. Pharmacy Technicians/<br>5. (pharmacy or pharmacies or drugstore or "drug store").mp. [mp=title, book title, abstract, original title, name of substance word, subject heading word, floating sub-heading word, keyword heading word, organism supplementary concept word, protocol supplementary concept word, rare disease supplementary concept word, unique identifier, synonyms, population supplementary concept word, anatomy supplementary concept word]<br>6. pharmacist*.mp. [mp=title, book title, abstract, original title, name of substance word, subject heading word, floating sub-heading word, keyboard heading word, organism supplementary concept word, protocol supplementary concept word, rare disease supplementary concept word, unique identifier, synonyms, population supplementary concept word, anatomy supplementary concept word]<br>7. 1 or 2 or 3 or 4 or 5 or 6<br>8. community pharmacy services/<br>9. {communit* adj5 pharmac*).mp. [mp=title, book title, abstract, original title, name of substance word, subject heading word, floating sub-heading word, keyboard heading word, organism supplementary concept word, protocol supplementary concept word, rare disease supplementary concept word, unique identifier, synonyms, population supplementary concept word, anatomy supplementary concept word]<br>10. 8 or 9<br>11. professional role/ or "scope of practice"/<br>12. "scope of practice".mp. [mp=title, book title, abstract, original title, name of substance word, subject heading word, floating sub-heading word, keyboard heading word, organism supplementary concept word, protocol supplementary concept word, rare disease supplementary concept word, unique identifier, synonyms, population supplementary concept word, anatomy supplementary concept word]<br>13. "practice scope".mp. [mp=title, book title, abstract, original title, name of substance word, subject heading word, floating sub-heading word, keyboard heading word, organism supplementary concept word, protocol supplementary concept word, rare disease supplementary concept word, unique identifier, synonyms, population supplementary concept word, anatomy supplementary concept word]<br>14. pharmacy based.mp.<br>15. (implement* adj2 practice).mp.<br>16. (role or roles).mp. [mp=title, book title, abstract, original title, name of substance word, subject heading word, floating sub-heading word, keyboard heading word, organism supplementary concept word, protocol supplementary concept word, rare disease supplementary concept word, unique identifier, synonyms, population supplementary concept word, anatomy supplementary concept word]<br>17. 11 or 12 or 13 or 14 or 15 or 16<br>18. exp clinical study/<br>19. intervention*.mp. [mp=title, book title, abstract, original title, name of substance word, subject heading word, floating sub-heading word, keyboard heading word, organism supplementary concept word, protocol supplementary concept word, rare disease supplementary concept word, unique identifier, synonyms, population supplementary concept word, anatomy supplementary concept word]

	20. randomized controlled trial.pt.<br>21. controlled clinical trial.pt.<br>22. (randomized or randomised).mp. or randomly.ab. [mp=title, book title, abstract, original title, name of substance word, subject heading word, floating sub-heading word, keyboard heading word, organism supplementary concept word, protocol supplementary concept word, rare disease supplementary concept word, unique identifier, synonyms, population supplementary concept word, anatomy supplementary concept word]<br>23. clinical trials as topic.sh.<br>24. trial.ti.<br>25. 19 or 20 or 21 or 22 or 23 or 24<br>26. 7 and 10 and 17 and 25<br>27. limit 26 to (english language and yr="2013-Current")
**Embase (OVID)**	pharmacist/<br>"pharmacy (shop)"/<br>pharmacy technician/<br>(pharmacy or pharmacies or "drug store" or drugstore).mp.<br>pharmacist*.mp.<br>1 or 2 or 3 or 4 or 5<br>community pharmacist/<br>(communit* adj5 pharmac*).mp.<br>7 or 8<br>professional standard/ or "scope of practice"/<br>pharmacy based.mp.<br>(implement* adj2 practice).mp.<br>"scope of practice".mp.<br>"practice scope".mp.<br>(role or roles).mp.<br>10 or 11 or 12 or 13 or 14 or 15<br>exp clinical study/<br>intervention*.mp.<br>randomized controlled trial/<br>controlled clinical study/<br>randomized.mp. or randomised.ab. [mp=title, abstract, heading word, drug trade name, original title, device manufacturer, drug manufacturer, device trade name, keyword heading word, floating subheading word, candidate term word]<br>randomly.ab.<br>"clinical trial (topic)"/<br>trial.ti.<br>17 or 18 or 19 or 20 of 21 or 22 or 23 or 24<br>6 and 9 and 16 and 25<br>limit 29 to (english language and yr="2013-Current")
**Emcare (OVID)**	1. pharmacist/<br>2. "pharmacy (shop)"<br>3. pharmacy technician/<br>4. (pharmacy or pharmacies or "drug store" or drugstore).mp.<br>5. pharmacist*.mp.<br>6. 1 or 2 or 3 or 4 or 5<br>7. community pharmacist/<br>8. (communit* adj5 pharmac*).mp.<br>9. 7 or 8

	10. professional standard/<br>11. “scope of practice”/<br>12. “scope of practice”.mp. [mp=title, abstract, heading word, drug trade name, original title, device manufacturer, drug manufacturer, device trade name, keyword heading word]<br>13. practice scope.mp. [mp=title, abstract, heading word, drug trade name, original title, device manufacturer, drug manufacturer, device trade name, keyword heading word]<br>14. (role or roles).mp. [mp=title, abstract, heading word, drug trade name, original title, device manufacturer, drug manufacturer, device trade name, keyword heading word]<br>15. pharmacy based.mp. [mp=title, abstract, heading word, drug trade name, original title, device manufacturer, drug manufacturer, device trade name, keyword heading word]<br>16. (implement* adj2 practice).mp. [mp=title, abstract, heading word, drug trade name, original title, device manufacturer, drug manufacturer, device trade name, keyword heading word]<br>17. 10 or 11 or 12 or 13 or 14 or 15 or 16<br>18. clinical study/<br>19. intervention*.mp.<br>20. randomized controlled trial.<br>21. controlled clinical trial/<br>22. (randomized or randomised).mp. [mp=title, abstract, heading word, drug trade name, original title, device manufacturer, drug manufacturer, device trade name, keyword heading word]<br>23. randomly.ab.<br>24. “clinical trial (topic)”/<br>25. trial.ti.<br>26. 10 or 19 or 20 or 21 or 22 or 23 or 24 or 25<br>27. 6 and 9 and 17 and 26<br>28. limit 27 to yr=”2013-Current”
**CINAHL Complete**	S1. (MH “Pharmacists”) OR (MH “Pharmacy Technicians”)<br>S2. (pharmacy or pharmacies or drugstore or “drug store”)<br>S3. pharmacist*<br>S4. (MH “Pharmacy Service+”)<br>S5. S1 or S2 or S3 or S4<br>S6. (MH “Community Role”)<br>S7. (communit* w4 pharmac*)<br>S8. S6 or S7<br>S9. (MH “Scope of Practice”)<br>S10. (MH “Professional Role”)<br>S11. “implement* w1 practice”<br>S12. “practice scope” OR “pharmacy based” OR “scope of practice”<br>S13. (role or roles)<br>S14. S8 or S9 or S10 or S11 or S12<br>S15. “clinical study”<br>S16. (MH “Intervention Trials”)<br>S17. MH double-blind studies OR MH single-blind studies OR MH random assignment OR MH pretest-posttest design OR MH cluster sample OR MH crossover design OR MH comparative studies<br>S18. intervention*<br>S19. AB randomly<br>S20. TI trial<br>S21. PT randomized controlled trial

	S22. randomized or randomised<br>S23. (MH "Clinical Trials")<br>S24. S14 OR S15 OR S16 OR S17 OR S18 OR S19 OR S20 OR S21 OR S22<br>S25. S5 AND S8 AND S14 AND S17 AND S24
**Scopus**	((TITLE-ABS-KEY (rct)) OR (TITLE-ABS-KEY ("random* control* trial*")) OR (TITLE-ABS-KEY ("clinical study" OR intervention*))) AND ((TITLE-ABS-KEY ("scope of practice")) OR (TITLE-ABS-KEY ("practice scope")) OR ("pharmacy based") OR ("implement* w/1 practice") OR (TITLE-ABS-KEY ((role OR roles)))) AND ((TITLE-ABS-KEY ((pharmacy OR pharmacies))) OR (TITLE-ABS-KEY (pharmacist*))) AND (TITLE-ABS-KEY ((communit* W/4 pharmac*))) AND (LIMIT-TO (PUBYEAR , 2024) OR LIMIT-TO (PUBYEAR , 2023) OR LIMIT-TO (PUBYEAR , 2022) OR LIMIT-TO (PUBYEAR , 2021) OR LIMIT-TO (PUBYEAR , 2020) OR LIMIT-TO (PUBYEAR , 2019) OR LIMIT-TO (PUBYEAR , 2018) OR LIMIT-TO (PUBYEAR , 2017) OR LIMIT-TO (PUBYEAR , 2016) OR LIMIT-TO (PUBYEAR , 2015) OR LIMIT-TO (PUBYEAR , 2014) OR LIMIT-TO (PUBYEAR , 2013)) AND (LIMIT-TO (LANGUAGE , "english"))
**Web of Science**	**"clinical study" OR intervention* OR rct OR "random* control* trial*"** (Topic) and **"scope of practice" OR "practice scope" OR (role or roles) OR "pharmacy based" OR "implement* NEAR/1 practice"** (Topic) and **(communit* NEAR/4 pharmac*)** (Topic) and **pharmacy or pharmacies or drugstore or "drug store" OR pharmacist*** (Topic) and **1 and 2 and 3 and 4** (All Fields) and **1 and 2 and 3 and 4 and 2024 or 2023 or 2022 or 2021 or 2020 or 2019 or 2018 or 2017 or 2016 or 2015 or 2014 or 2013 (Publications Years) and English** (All Fields)

SUPPLEMENTARY TABLE S2: CHARACTERISTICS TABLE

TITLE	AUTHORS, YEAR, COUNTRY	SETTING	AIM	STUDY DESIGN AND LENGTH OF THE INTERVENTION	INTERVENTION GROUP	CONTROL/STANDARD GROUP	STRENGTHS	LIMITATIONS
Virtual Coaching Delivered by Pharmacists to Prevent COVID-19 Transmission	Abdel-Qader et al, 2022<br><br>Jordan	49 community pharmacies in four different Jordanian regions	To assess whether pharmacist-based virtual health coaching sessions may increase the fraction of study participants who practised healthy social behaviours, to test whether this model can increase the public acceptance of COVID-19 vaccines, and to measure whether these behaviours may actually prevent contracting COVID-19.	This RCT was conducted between January 03 and March 25, 2021	12 pharmacist-based virtual coaching sessions; a session per week.	No coaching.	Pharmacists were trained that knowledge-based coaching alone would not enhance behavioural change, and so knowledge should be adapted to each participants' lifestyle.	Findings were subjected to participants' bias, as self-reporting for outcome assessment was used<br><br>Control participants were not followed to track if they got the virus or not.

TITLE	AUTHORS, YEAR, COUNTRY	SETTING	AIM	STUDY DESIGN AND LENGTH OF THE INTERVENTION	INTERVENTION GROUP	CONTROL/STANDARD GROUP	STRENGTHS	LIMITATIONS
The Effectiveness of Pharmacist Interventions on Cardiovascular Risk in Adult Patients with Type 2 Diabetes: The Multicentre Randomized Controlled R$_x$EACH Trial	Al Hamarneh et al, 2017<br><br>Canada	56 community pharmacies across the province Alberta	To evaluate the effect of pharmacist case finding and intervention program on estimated cardiovascular (CV) risk in patients with diabetes	Randomised controlled trial between January 2014 and June 2015	Medication Therapy Management consultations with community pharmacist:<br>1. Patient assessment: blood-pressure measurement, weight and height measurements;<br>2. Laboratory assessment: A1C level, lipid profile, kidney function and<br>3. Individualised CV risk assessment: risk calculation, education and discussion of risk	Usual pharmacist and physician care, with no specific interventions	Subgroup analysis contributes to the high level of evidence of effective pharmacist interventions in improving glycaemic control, medication adherence, diabetes awareness, morbidity outcomes	The 3-month follow-up period may be short; effects of the intervention may be short-lived<br><br>Blinding was not possible due to the nature of the intervention

TITLE	AUTHORS, YEAR, COUNTRY	SETTING	AIM	STUDY DESIGN AND LENGTH OF THE INTERVENTION	INTERVENTION GROUP	CONTROL/STANDARD GROUP	STRENGTHS	LIMITATIONS
Use of effective contraception following provision of the progestogen-only pill for women presenting to community pharmacies for emergency contraception (Bridge-It): a pragmatic cluster-randomised crossover trial	Cameron et al, 2020<br><br>UK	29 community pharmacies in the regions of: London (south and central), Lothian (Edinburgh and region), and Tayside (Dundee and region)	To determine whether the bridging-supply intervention resulted in increased use of subsequent effective contraception (hormonal or intrauterine) compared with the provision of emergency contraception alone.	This cluster-randomised cohort crossover trial was conducted between December 19 2017, and June 26 2019	Women requesting emergency contraception received three packets (q = 28) of the progestogen-only pill (75 microg desogestrel per day) at no cost, without a prescription (bridging-supply intervention).<br><br>Women also received a rapid access card that allowed for discussion and obtaining alternative effective contraception when presented to the local participating sexual and reproductive health clinic.	Women receiving emergency contraception from the community pharmacy (levonorgestrel 1.5 mg or 3 mg).	Appropriate sample size to demonstrate expected outcomes with sufficient power.<br><br>This study incorporated a mix of large chain and small independent community pharmacies that supply emergency contraception at a high volume and so the results are generalisable to UK pharmacies in which most emergency contraception is provided.<br><br>No differential loss to follow-up either between intervention groups or between recruitment periods.	Dispensing emergency contraception is timely and so asking pharmacists to participate in research through the recruitment of participants, provide further information and complete additional paperwork as part of a research study may be an added burden.<br><br>Recruitment took longer than anticipated and there were large differences between pharmacies in the number of women recruited and within pharmacies in the number recruited in each period<br><br>There was a substantial drop-out rate for the self-reported primary outcome of effective contraception use at 4 months

TITLE	AUTHORS, YEAR, COUNTRY	SETTING	AIM	STUDY DESIGN AND LENGTH OF THE INTERVENTION	INTERVENTION GROUP	CONTROL/STANDARD GROUP	STRENGTHS	LIMITATIONS
A randomised controlled trial of pharmacist-led therapeutic carbohydrate and energy restriction in type 2 diabetes	Durrer et al, 2019<br><br>Canada	12 community pharmacies across southern southern British Columbia	A very low-carbohydrate, low-calorie diet could lessen the need for glucose-lowering agents and improve cardiovascular health when compared to treatment-as-usual.	12-week pragmatic, parallel-group RCT between 7 July 2017 and 1 April 2019	Community pharmacist-based therapeutic carbohydrate restriction (Pharm-TCR) group: commercial weight lost diet plan - reduced-carbohydrate, energy-restricted, protein meals and snacks.<br><br>Weekly check-ins with lifestyle coach and pharmacist to monitor progress	Standard medication advice by pharmacist and information leaflets on diet and lifestyle	Pragmatic nature of the trial may have demonstrated potential insights that may be of benefit for the implementation of similar interventions in the community pharmacy setting<br><br>Using a standardised medication deprescription plan assisted in the consistent implementation of the Pharm-TCR intervention	The attrition rate (13.56% for the intervention group and 12.71% for the control group) suggests that this type of intervention delivered in the communitymight not be suitable for everyone.<br><br>Pharm-TCR intervention provided free of charge, and so this may impact the interpretation of the findings.

TITLE	AUTHORS, YEAR, COUNTRY	SETTING	AIM	STUDY DESIGN AND LENGTH OF THE INTERVENTION	INTERVENTION GROUP	CONTROL/STANDARD GROUP	STRENGTHS	LIMITATIONS
Pharmacist's interventions improve time in therapeutic range of elderly rural patients on warfarin therapy: a randomized trial	Falamic et al, 2018<br><br>Croatia	A community pharmacy in Donji Miholjac, province of Slavonia	To evaluate the effect of a community pharmacist's interventions on the quality of anticoagulation practice in elderly rural patients receiving warfarin	RCT was facilitated from May 2015 to January 2017	All participants were followed up monthly for a period of 6 months.<br><br>Repeated education and a follow-up were given to the participants in the intervention group, and their GPs were contacted to amend the warfarin dose or to avoid drug interactions.	Standard anticoagulation GP-managed care.<br><br>Monthly follow-up visit	Double-blinded design reduced researcher bias.	Findings are geographically limited as only one community pharmacy recruiting and providing service was employed in this study<br><br>Study participants had a defined therapeutic range of INR 2-3 therefore the results are not generalisable for the wider population.<br><br>Study participants may not represent elderly rural patients in other countries because of socioeconomic, cultural and other differences.<br><br>Knowledge of patients about warfarin was not measured.

TITLE	AUTHORS, YEAR, COUNTRY	SETTING	AIM	STUDY DESIGN AND LENGTH OF THE INTERVENTION	INTERVENTION GROUP	CONTROL/STANDARD GROUP	STRENGTHS	LIMITATIONS
Improving care transitions through medication therapy management: A community partnership to reduce readmissions in multiple health-systems	Heaton et al, 2018<br><br>USA	Hybrid (60 community pharmacies within a supermarket pharmacy chain and six hospitals), Cincinnati	Primary objective was to measure the effect of a pharmacist-directed medication therapy program on 30-day post-discharge readmission rates to hospital<br><br>Secondary study objectives were to identify the number and types of pharmacist interventions, to determine the impact of the intervention on medication nonadherence, and to measure patient satisfaction with the intervention.	Assisting the transfer of care from the inpatient setting to the community setting across the entire Cincinnati area.<br><br>The study period was May 1, 2015, to August 2, 2017.	Within 1 week of discharge, the Continuity of Care Document (CCD) was created and sent to the call centre. The call centre scheduled appointments with a Kroger-employed pharmacist at the patient's preferred location.<br><br>Kroger pharmacists received implementation support.<br><br>The CCD was used to base the MTM appointment, including medication reconciliation, comprehensive medication review, , and education on new medication or diagnoses. Pharmacists also provided self-management education and set health-related goals.<br><br>The pharmacist counselled on appropriate action to prevent further decline and to prevent hospitalisation.	The control group did not provide the (medication therapy management) MTM service, unlike the intervention group.	Most of the interventions were deemed acceptable by patients and physicians.	The intent-to-treat (ITT) cohort at baseline displayed demographic differences between the control and intervention groups.<br><br>The study was carried-out in a specific region of the Midwest and so findings may not be generalisable.<br><br>Many hospitals and insurance plan are introducing programs to target Medicare patients. Consequently, the readmission rates in the control group could have been disproportionately affected by coexisting readmission reduction initiatives compared with the intervention group, which included younger patients who may not be eligible for other transition-of-care programs.<br><br>The intervention group had a significantly higher number of Medicare patients, who are more likelyto have more complex socioeconomic issues that can impact care.<br><br>The ability to measure the impact of the pharmacists' interventions was limited because of small sample sizes.

					A folder was provided that included a personal medication list and medication action plan, a list of upcoming appointments, and to facilitate self-monitoring, patients received weight, blood pressure, blood sugar, or other appropriate logs.<br><br>One week after the initial MTM visit, pharmacists communicated all medication recommendations to the patient's primary care physician.			

TITLE	AUTHORS, YEAR, COUNTRY	SETTING	AIM	STUDY DESIGN AND LENGTH OF THE INTERVENTION	INTERVENTION GROUP	CONTROL/STANDARD GROUP	STRENGTHS	LIMITATIONS
The effectiveness of pharmacist-based coaching in improving breast cancer-related health behaviours: A randomised controlled trial	Ibrahim et al, 2021<br><br>Egypt	Community pharmacy	To study the effectiveness for pharmacist-led coaching in BC-related health behaviours and knowledge in females, and to measure the comfort level of the intervention.	This was an RCT facilitated in Egypt between January 07 and June 01 of 2021	Females enrolled in a face-to-face health coaching program implemented by community pharmacists	Females not part of the coaching program.	Study pharmacists received comprehensive online training on theoretical and practical aspects of health coaching and in recruiting participants.	Self-reporting as an outcome assessment method may introduce bias to findings.<br><br>Follow-up was up to three months post-coaching, which may not be sufficient to measure the impact of the intervention.<br><br>Study findings may not be generalisable to all females in Egypt as this study was facilitated in specific cities<br><br>Competency limitations of coaches may be due to period of training that was received.

TITLE	AUTHORS, YEAR, COUNTRY	SETTING	AIM	STUDY DESIGN AND LENGTH OF THE INTERVENTION	INTERVENTION GROUP	CONTROL/STANDARD GROUP	STRENGTHS	LIMITATIONS
Effect of a Community Pharmacist-Delivered Diabetes Support Program for Patients Receiving Specialty Medicare Care: A Randomised Controlled Trial	Jahangard-Rafsanjani et al, 2014<br><br>Iran	Nemooneh-Taleghani Community Pharmacy, affiliated with the College of Pharmacy, Tehran University of Medical Sciences, Iran	To assess the efficacy of a community pharmacist-delivered diabetes intervention for patients receiving specialty medical care in Iran	Parallel-group RCT<br><br>Participants were recruited between March 2012 and April 2013.	Community pharmacist educated patients about medications, clinical goals, and self-monitoring of blood glucose for 5 months, and referred to the primary-care physician when required.	Usual care delivered by primary-care physician<br><br>Baseline tests were retrieved by the community pharmacist at the point of recruitment<br><br>Study participants received a final assessment at the end of the study. Pharmacist briefed participants on diabetes self-care and helped them locate an appropriate diabetes education program.<br><br>Study pharmacists noted the number of physician visits and the drug therapy modifications during the study period.	This study was a rigorous on the effect of diabetes education and support program in a middle-income country.<br><br>Collaborative study between a local community pharmacy and diabetes specialty care.<br><br>All A1c assessments were performed by 1 laboratory to minimise bias.	Data from 85 patients were available for analysis, which was less than the targeted sample size.

TITLE	AUTHORS, YEAR, COUNTRY	SETTING	AIM	STUDY DESIGN AND LENGTH OF THE INTERVENTION	INTERVENTION GROUP	CONTROL/STANDARD GROUP	STRENGTHS	LIMITATIONS
Effects of Telephone Counseling Intervention by Pharmacists (TelCIP) on Medication Adherence; Results of a Cluster Randomized Trial	Kooij et al, 2016<br><br>Netherlands	53 community pharmacies	To assess the effect of a pharmacist telephone counselling intervention on patients' medication adherence	This RCT was conducted in 53 community pharmacies between October 2010 and March 2013	Study participants were contacted via telephone by a pharmacist between 7 and 21 days after the first prescription.<br><br>The call was supported by a pre-tested interview guide aimed at addressing: (1) need for additional information; (2) actual medication use; (3) practical barriers ; (4) perceptual barriers	Study participants received both written and verbal information at prescription initiation (maximum of 2 week supply)<br><br>At the first refill, participants are asked about their experiences with the medication. If necessary, additional information or counselling should be provided.	Study included four different medication classes.<br><br>The pragmatic design of the trial and large number of study pharmacies contributes to the generalizability of the results.<br><br>A relatively high proportion of study participants received the intervention.<br><br>This intervention included study participants irrespective if they returned for a refill or not. This means that patients who decided not to initiate or to discontinue were approached. This is relevant as a high number of patients tend to discontinue therapy in the first weeks.<br><br>Study participants unable to attend the pharmacy were also included.<br><br>Study contamination was reduced using a cluster designimplementing standardised training, providing medication class specific interview guidelines and treatment manual, and the requirement for pharmacists to complete a self-report questionnaire for every selected study participant.	In some studies that were reviewed pre-empting this RCT, patients had a verified diagnosis of depression. This is absent in the current study and it is unknown if the medication was prescribed for depression or other indications such as anxiety disorders.

TITLE	AUTHORS, YEAR, COUNTRY	SETTING	AIM	STUDY DESIGN AND LENGTH OF THE INTERVENTION	INTERVENTION GROUP	CONTROL/STANDARD GROUP	STRENGTHS	LIMITATIONS
Pharmacy diabetes screening trial (PDST): Outcomes of a national clustered RCT comparing three screening methods for undiagnosed type 2 diabetes (T2DM) in community pharmacy	Krass et al, 2022<br><br>Australia	339 community pharmacies	To compare the effectiveness of three pharmacy-based screening methods for type 2 diabetes (T2DM)	Three-arm clustered randomised controlled trial carried out in community pharmacies between March and October 2017	Diabetes screening in an Australian community pharmacy setting:<br>1. AUSDRISK alone (Group A)<br>2. AUSDRISK followed by a POC HbA1c test (Group B)<br>3. AUSDRISK followed by a POC scBGT (Group C)	Calculation of a risk score using the 10-item Australian Type 2 Diabetes Risk Assessment (AUSDRISK)<br><br>AUSDRUSK is a validated risk assessment tool that predicts the likeliness of developing diabetes within 5 years based on the presenting set of known risk factors	Compared to most other pharmacy screening studies that rely on participating self-report for diagnostic outcomes, in this trial, confirmation of most diagnoses of diabetes and prediabetes were established either from medical records or pathology test results<br><br>To account for potential bias, the ITT analysis included all screening participants in the final analysis, regardless of non-compliance or withdrawal<br><br>This trial included pharmacies representing a wide range of local demographics and regions across Australia<br><br>To accommodate pharmacy withdrawals post saturation of the postcodes, a convenience sample of pharmacies with prior trial or known high-service level experience was recruited and randomly allocated to each of the trial arms	Small proportion of referred screening participants for whom follow-up attempts with either the patient, pathology laboratory and GP failed to confirm an outcome

TITLE	AUTHORS, YEAR, COUNTRY	SETTING	AIM	STUDY DESIGN AND LENGTH OF THE INTERVENTION	INTERVENTION GROUP	CONTROL/STANDARD GROUP	STRENGTHS	LIMITATIONS
A cluster randomised control trial to evaluate the effectiveness and cost-effectiveness of the Italian medicines use review (I-MUR) for asthma patients	Manfrin et al, 2017<br><br>Italy	Community pharmacies from 15 out of 20 Italian regions	To assess the effectiveness and cost-effectiveness of Medicines Use Reviews implemented by community pharmacists relating to asthma management	This clustermulti-centre, RCT in adult patients with asthma was carried-out between September 2014 and July 2015.	Systematic and structured interview facilitated in a private consulting room which addressed asthma symptoms, medicines used, attitudes towards medicines and adherence.<br><br>Pharmacists were made to identify pharmaceutical care issues (PCIs) which may influence optimal medicines use and therefore asthma control and counsel patients and recommendations to their GP, when required.	To minimise study bias this cluster RCT had a phased intervention, with control clusters delivering the intervention after the primary end-point was complete<br><br>As a result, all pharmacists (intervention and control) provided the intervention to study participants but at different time points during the study.	First RCT in Italy to evaluate a community pharmacist-based intervention.<br><br>This study adopted the ACT score as a measure of asthma control, which encouraged measurement of both effectiveness and cost-effectiveness (by translating ATC scores into QALYs) of the intervention<br><br>Power attained by the study was 90%	Study pharmacists selected participants and entered the data collected onto the electronic template, which is a possible sources of bias.<br><br>The assessment of adherence was not done so using a validated tool.<br><br>The initial follow-up period before the usual care group received the intervention was only 3 months. Therefore, it had to be assumed that no change in ACT control from T3 onwards for the economic analysis.<br><br>Secondary data rather than primary data was collected on cost and utility, which were derived from old estimates were old estimates, actualised at 2015.

TITLE	AUTHORS, YEAR, COUNTRY	SETTING	AIM	STUDY DESIGN AND LENGTH OF THE INTERVENTION	INTERVENTION GROUP	CONTROL/STANDARD GROUP	STRENGTHS	LIMITATIONS
An Interventional Call-Back Service to Improve Appropriate Use of Antibiotics in Community Pharmacies	Paravattil et al, 2021<br><br>Qatar	6 community pharmacies	To investigate the appropriateness of antibiotic supply in community pharmacy settings while implementing a call-back service to determine adherence and symptom resolvement among patients prescribed an antibiotic.	A multicentred interventional study	Interventional call-back service	The counselling provided to control patients is at the discretion of the pharmacist.	The included community pharmacies were representative of a diverse sample of residents residing in Qatar.	Due to the limited collaboration between pharmacists and prescribers, monitoring prescribing patterns may be difficult. Lack of reimbursement or access to patient health records are barriers to participating in antimicrobial stewardship activities linked to prescribing practices.

TITLE	AUTHORS, YEAR, COUNTRY	SETTING	AIM	STUDY DESIGN AND LENGTH OF THE INTERVENTION	INTERVENTION GROUP	CONTROL/STANDARD GROUP	STRENGTHS	LIMITATIONS
Randomised Trial of the Effect of Pharmacist Prescribing on Improving Blood Pressure in the Community: The Alberta Clinical Trial in Optimising Hypertension (RxACTION)	Tsuyuki et al, 2015<br><br>Canada	Community pharmacies, primary care teams or hospitals in 23 communities in the province of Alberta	To establish the impact of pharmacist prescribing on blood pressure (BP) control	A patient-level, RCT spanning a total of 6-months<br><br>This study concluded in May 2013	Following-up of BP and cardiovascular risk, patient-education on hypertension, prescribing of antihypertensive agents, laboratory marker assessment, and monthly check-ins for 6 months.	A card for BP recording, written information, and usual care from their pharmacist and physician.	To reduce the impact of white coat-syndrome on potentially elevating hypertension, a BP device was used for all study measurements, and pharmacists left the room when these measurements were taken to further manage patient anxiety.	Pharmacists nor patients could be blinded in this study to which patients were allocated.<br><br>Study participants were seen more often by the pharmacist if randomised to the intervention-arm, which may have resulted in the BP reduction observed attributed to reduced white coat hypertension over time or regression to the mean.<br><br>Possibility of contamination amongst the usual care group because cluster randomisation was not used in this trial.<br><br>The target sample of 340 patients was not achieved.

TITLE	AUTHORS, YEAR, COUNTRY	SETTING	AIM	STUDY DESIGN AND LENGTH OF THE INTERVENTION	INTERVENTION GROUP	CONTROL/STANDARD GROUP	STRENGTHS	LIMITATIONS
A randomised trial of a community-based approach to dyslipidaemia management: Pharmacist prescribing to achieve cholesterol targets (RxACT Study)	Tsuyuki et al, 2016<br><br>Canada	Community pharmacies in the province of Alberta	The primary objective of this study was to assess the impact of participant assessment, care plan, education/counselling, prescribing/titration of lipid-lowering medications and close monitoring on the fraction of participants achieving target LDL-c levels as defined by the 2009 Canadian Cardiovascular Society (CCS) dyslipidaemia guidelines.<br><br>Secondary objectives included establishing the impact of the intervention on the difference in change in LDL-c and apolipoprotein-B (Apo-B) between groups at 6 months.	This was an RCT of pharmacist intervention, with the unit of randomisation between December 2011 and July 2013.	Pharmacist-led dyslipidaemia management, including assessment of cardiovascular risk, review of LDL-c, prescribing of lipid-lowering agents, health behaviour interventions and follow-up every 6 weeks for 6 months.	Participants received a copy of their lab results, a leaflet on cardiovascular disease and standard care from their pharmacist and physician.	Pharmacists were invited to apply 'case finding' as part of the recruitment strategy<br><br>Participants were randomised in a 1:1 ratio to allow allocation concealment<br><br>Implemented on a wider-scale, this study may assist counteract 'clinical inertia' and help more patients to achieve lipid targets	Blinding was not possible in this study.<br><br>It was not possible to measure change in apo-B, one of the secondary outcomes, as it was not consistently collected<br><br>Since approximately 25% of Alberta-basedcommunity-pharmacists currently have additional prescribing authority, it is possible that the study pharmacists are not representative of the wider population of practicing pharmacists.

SUPPLEMENTARY TABLE S3: OUTCOMES TABLE

TOPIC	TITLE	EFFICACIOUS (Y/N)	EFFICACY OF THE INTERVENTION	POTENTIAL TO CHANGE COMMUNITY PHARMACY SCOPE OF PRACTICE IDENTIFIED (Y/N)	CHANGE TO COMMUNITY PHARMACY SCOPE OF PRACTICE	AUTHORS AND YEAR
**COLLABORATIVE CARE**	Virtual Coaching Delivered by Pharmacists to Prevent COVID-19 Transmission	Y	Pharmacist-based virtual health coaching could be a potential strategy to increase the proportion of behaviours that could curtail the spread of COVID-19.<br><br>The findings of this study indicate that pharmacist-induced health coaching improved the proportion of study participants who were willing to administer the COVID-19 vaccine.	Y	Pharmacist-induced virtual health coaching may be a potential means to improve the proportion of behaviours that could control the spread of COVID-19.	Abdel-Qader et al, 2022

TOPIC	TITLE	EFFICACIOUS (Y/N)	EFFICACY OF THE INTERVENTION	POTENTIAL TO CHANGE COMMUNITY PHARMACY SCOPE OF PRACTICE IDENTIFIED (Y/N)	CHANGE TO COMMUNITY PHARMACY SCOPE OF PRACTICE	AUTHORS AND YEAR
**COLLABORATIVE CARE**	Use of effective contraception following provision of the progestogen-only pill for women presenting to community pharmacies for emergency contraception (Bridge-It): a pragmatic cluster-randomised crossover trial	Y	The bridging-intervention, that is, supply of the progestogen-only pill (75 microg desogestrel) with the emergency contraceptive pill (levonorgestrel 1.5 mg or 3 mg) from a community pharmacist, in conjunction with a rapid access referral to a sexual and reproductive health clinic, results in a clinically significant increase in subsequent use of effective contraception.	Y	Pharmacist supply of the progestogen-only-pill (75 microg desogestrel, q = 84 tablets) after the use of the emergency contraceptive pill (levonorgestrel 1.5 mg or 3 mg) without requiring a prescription.<br><br>The bridging-intervention if implemented on a large-scale may prevent unintended pregnancies after the use of the emergency contraceptive pill (levonorgestrel 1.5 mg or 3 mg).	Cameron et al, 2020

TOPIC	TITLE	EFFICACIOUS (Y/N)	EFFICACY OF THE INTERVENTION	POTENTIAL TO CHANGE COMMUNITY PHARMACY SCOPE OF PRACTICE IDENTIFIED (Y/N)	CHANGE TO COMMUNITY PHARMACY SCOPE OF PRACTICE	AUTHORS AND YEAR
**COLLABORATIVE CARE**	Improving care transitions through medication therapy management: A community partnership to reduce readmissions in multiple health-systems	Y	This study successfully implemented a transition-of-care program collaborating multiple health systems and community pharmacies<br><br>Hospital readmissions were approximately reduced by 9% for patients who kept their appointments with a pharmacist as a result of the intervention<br><br>Pharmacists identified around six interventions per patient	Y	This study successfully introduceda large-scale transition-of-care program collaborating between multiple health systems and community pharmacies that reduced hospital readmissions by approximately 9% for patients who attended a scheduled appointment with a pharmacist.<br><br>This RCT demonstrated that pharmacists can improve positive patient outcomes and therefore expanding scope of practice to address this gap in service when transitioning from the hospital-setting to the community-setting may optimise the role of the pharmacist.	Heaton et al, 2018

TOPIC	TITLE	EFFICACIOUS (Y/N)	EFFICACY OF THE INTERVENTION	POTENTIAL TO CHANGE COMMUNITY PHARMACY SCOPE OF PRACTICE IDENTIFIED (Y/N)	CHANGE TO COMMUNITY PHARMACY SCOPE OF PRACTICE	AUTHORS AND YEAR
**COLLABORATIVE CARE**	The effectiveness of pharmacist-based coaching in improving breast cancer-related health behaviours: A randomised controlled trial	Y	Forming a coaching program for females in community pharmacies resulted in greater proportions of several BC-related health behaviours including high physical activity, healthy diet, practicing breastfeeding, BSE, and the willingness to consult healthcare professionals on BC issues.	Y	An expanded scope of pharmacist-based health coaching can improve breast cancer (BC) related health behaviours and knowledge in females with acceptable comfort levels.	Ibrahim et al, 2021

TOPIC	TITLE	EFFICACIOUS (Y/N)	EFFICACY OF THE INTERVENTION	POTENTIAL TO CHANGE COMMUNITY PHARMACY SCOPE OF PRACTICE IDENTIFIED (Y/N)	CHANGE TO COMMUNITY PHARMACY SCOPE OF PRACTICE	AUTHORS AND YEAR
**MEDICATION ADHERENCE**	The Effectiveness of Pharmacist Interventions on Cardiovascular Risk in Adult Patients with Type 2 Diabetes: The Multicentre Randomized Controlled R_xEACH Trial	Y	This study found that community pharmacy-based case-finding and community pharmacist intervention was associated with significant improvement in cardiovascular risk, individual cardiovascular risk factors, and treatment regimens when compared to usual practice.<br><br>This study highlights the value of pharmacist prescribing in that significant improvement in diabetes, hypertension and dyslipidaemia treatments in the intervention-group were observed compared to control.	Y	Comprehensive Medication Therapy Management (MTM) consultations (on an ongoing basis) in a community pharmacy setting encompassing patient assessment, individualised CV risk assessment, treatment recommendations, regular communication with the treating physician to inform patient updates, and in particular, laboratory assessment and pharmacist prescribing, supplements an expanded scope of community pharmacy practice.	Al Hamarneh et al, 2017

TOPIC	TITLE	EFFICACIOUS (Y/N)	EFFICACY OF THE INTERVENTION	POTENTIAL TO CHANGE COMMUNITY PHARMACY SCOPE OF PRACTICE IDENTIFIED (Y/N)	CHANGE TO COMMUNITY PHARMACY SCOPE OF PRACTICE	AUTHORS AND YEAR
**MEDICATION ADHERENCE**	Effects of Telephone Counseling Intervention by Pharmacists (TelCIP) on Medication Adherence; Results of a Cluster Randomized Trial	N	Overall, no effect of the intervention was found.<br><br>Results suggest that adherence improved for patients starting with RAS-inhibitors, statins or bisphosphonates. For antidepressants, no significant effect was found.<br><br>The counselling may have assisted study participants to make an informed decision whether or not to continue treatment, although the effect on adherence may have been the same.	Y	There is potential in expanding community pharmacist scope of practice to implement follow-up phone calls, although by large, no effect for on adherence in patients initiating antidepressants were found.	Kooij et al, 2016

TOPIC	TITLE	EFFICACIOUS (Y/N)	EFFICACY OF THE INTERVENTION	POTENTIAL TO CHANGE COMMUNITY PHARMACY SCOPE OF PRACTICE IDENTIFIED (Y/N)	CHANGE TO COMMUNITY PHARMACY SCOPE OF PRACTICE	AUTHORS AND YEAR
**MEDICATION ADHERENCE**	An Interventional Call-Back Service to Improve Appropriate Use of Antibiotics in Community Pharmacies	Y	A community pharmacist call-back service is a simple and inexpensive intervention that can improve appropriate antibiotic use, particularly in relation to adherence.<br><br>The call-back and structured counselling intervention recorded higher antibiotic adherence rate when compared to the standard care group.	Y	In conjunction to the pharmacist's role as an educator, community pharmacists are essental in impacting prescribing habits amongst providers.<br><br>The study results found that educating patients on the adequate use of antibiotics either through comprehensive counselling or a call-back service improves adherence.<br><br>Community pharmacists require targeted and continuous education and training in antimicrobial stewardship. Their roles extend beyond patient counselling and so training should focus on the judicious prescribing of antibiotics.<br><br>Community pharmacists and general practitioners need to work collaboratively to reduce antibiotic misuse.	Paravattil et al, 2021

TOPIC	TITLE	EFFICACIOUS (Y/N)	EFFICACY OF THE INTERVENTION	POTENTIAL TO CHANGE COMMUNITY PHARMACY SCOPE OF PRACTICE IDENTIFIED (Y/N)	CHANGE TO COMMUNITY PHARMACY SCOPE OF PRACTICE	AUTHORS AND YEAR
**MEDICATION ADHERENCE**	Randomised Trial of the Effect of Pharmacist Prescribing on Improving Blood Pressure in the Community: The Alberta Clinical Trial in Optimising Hypertension (RxACTION)	Y	This study highlightssignificant reductions in systolic and diastolic BPs and significant improvements in the proportion of patients achieving recommended BP targets compared with usual pharmacist and physician care.<br><br>Pharmacist-induced prescribing in conjunction to usual care, results in a clinically significant reduction in BP and a substantial increase in the proportion of patients with initially uncontrolled hypertension achieving their target BP.	Y	Despite Alberta being the first Canadian jurisdiction to authorise pharmacists to independently prescribe antihypertensive drug therapy for patients, the Canadian provinces of Manitoba and New Brunswick have recently implemented similar legislation for pharmacists with specialty training and working in colloborative borative practice. Independent pharmacist prescribing has been in place in the United Kingdom since 2006.<br><br>This study backs an expanded community pharmacists' scope of practice to include medication management activities in an effort to address clinical inertia in hypertension management.	Tsuyuki et al, 2015

TOPIC	TITLE	EFFICACIOUS (Y/N)	EFFICACY OF THE INTERVENTION	POTENTIAL TO CHANGE COMMUNITY PHARMACY SCOPE OF PRACTICE IDENTIFIED (Y/N)	CHANGE TO COMMUNITY PHARMACY SCOPE OF PRACTICE	AUTHORS AND YEAR
**MEDICATION ADHERENCE**	A randomised trial of a community-based approach to dyslipidaemia management: Pharmacist prescribing to achieve cholesterol targets (RxACT Study)	Y	Independent community-pharmacist prescribing results in a clinically significant improvement to the proportion of patients achieving guideline treatment targets in the management of dyslipidaemia and a reduction in LDL-c levels.	Y	Independent community-pharmacist prescribing resulted in >3-fold more patients achieving target LDL-c levels. This may have major public health implications.	Tsuyuki et al, 2016

TOPIC	TITLE	EFFICACIOUS (Y/N)	EFFICACY OF THE INTERVENTION	POTENTIAL TO CHANGE COMMUNITY PHARMACY SCOPE OF PRACTICE IDENTIFIED (Y/N)	CHANGE TO COMMUNITY PHARMACY SCOPE OF PRACTICE	AUTHORS AND YEAR
**MEDICATION MANAGEMENT**	A randomised controlled trial of pharmacist-led therapeutic carbohydrate and energy restriction in type 2 diabetes	Y	The Pharm-TCR intervention was effective in lowering the need for glucose-lowering agents through complete discontinuation of medications and reduced medication effect score compared to TAU.	Y	Findings suggest that community pharmacists are a viable and innovative avenue for implementing short-term nutritional interventions for people with type 2 diabetes, particularly when medication management is a safety concern.	Durrer et al, 2019

TOPIC	TITLE	EFFICACIOUS (Y/N)	EFFICACY OF THE INTERVENTION	POTENTIAL TO CHANGE COMMUNITY PHARMACY SCOPE OF PRACTICE IDENTIFIED (Y/N)	CHANGE TO COMMUNITY PHARMACY SCOPE OF PRACTICE	AUTHORS AND YEAR
**MEDICATION MANAGEMENT**	Pharmacist's interventions improve time in therapeutic range of elderly rural patients on warfarin therapy: a randomized trial	Y	Community pharmacist's interventions significantly improved TTR in elderly rural patients in Croatia.	Y	Barriers in healthcare of rural patients could be bridged by community pharmacist's involvement.<br><br>These interventions may be implemented in a range of models and can potentially be an alternative to DOACs, especially in countries where DOACs are a big financial burden to patients.	Falamic et al, 2018

TOPIC	TITLE	EFFICACIOUS (Y/N)	EFFICACY OF THE INTERVENTION	POTENTIAL TO CHANGE COMMUNITY PHARMACY SCOPE OF PRACTICE IDENTIFIED (Y/N)	CHANGE TO COMMUNITY PHARMACY SCOPE OF PRACTICE	AUTHORS AND YEAR
**MEDICATION MANAGEMENT**	Effect of a Community Pharmacist-Delivered Diabetes Support Program for Patients Receiving Specialty Medicare Care: A Randomised Controlled Trial	N	This intervention improved self-care activity, medication adherence, and body mass index in study participants Although the amount of A1C reduction was higher in the intervention group, it was not statistically significant.	Y	Pharmacist's role in weight management is an area of continual developmentand may be incorporated with diabetes education programs in community pharmacy practice.<br><br>This study was largely impacted by evidence-based medication therapy rather than diabetes education.	Jahangard-Rafsanjani et al, 2014

TOPIC	TITLE	EFFICACIOUS (Y/N)	EFFICACY OF THE INTERVENTION	POTENTIAL TO CHANGE COMMUNITY PHARMACY SCOPE OF PRACTICE IDENTIFIED (Y/N)	CHANGE TO COMMUNITY PHARMACY SCOPE OF PRACTICE	AUTHORS AND YEAR
**MEDICATION MANAGEMENT**	Pharmacy diabetes screening trial (PDST): Outcomes of a national clustered RCT comparing three screening methods for undiagnosed type 2 diabetes (T2DM) in community pharmacy	Y	Implementing a stepwise approach using the AUSDRISK screening tool as a baseline measure, followed by a point-of-care (POC) test if the AUSDRISK score represents elevated risk and subsequent referral based on threshold values of HbA1c, FBG, or RBG, was a more effective means in identifying new cases of T2DM in a community pharmacy setting when compared to risk assessment alone.	Y	Whilst T2DM screening strategies are offered in community pharmacies to varying extents, the POC measure displays an expanded scope of community pharmacy practice. Risk assessment tools involving the calculation of a risk score alone, demonstrates usual care.	Krass et al, 2022

TOPIC	TITLE	EFFICACIOUS (Y/N)	EFFICACY OF THE INTERVENTION	POTENTIAL TO CHANGE COMMUNITY PHARMACY SCOPE OF PRACTICE IDENTIFIED (Y/N)	CHANGE TO COMMUNITY PHARMACY SCOPE OF PRACTICE	AUTHORS AND YEAR
**MEDICATION MANAGEMENT**	A cluster randomised control trial to evaluate the effectiveness and cost-effectiveness of the Italian medicines use review (I-MUR) for asthma patients	Y	Usual care in community pharmacy in Italy has little clinical input, and therefore are mainly a supply function, and so the patients were not used to a pharmacist providing information about optimising medicines use, which may have contributed to the effectiveness of the intervention.	Y - RCT resulted in *actual* change to community pharmacy scope of practice	This study demonstrates that the intervention was both effective and cost-effective.<br><br>The Italian Government have since implemented a change of community pharmacy practice, with the I-MUR being the first nationally funded cognitive pharmaceutical service in Italy.<br><br>This in turn supports the change from a largely logistical model to a more patient-centred and clinically-oriented role of the community pharmacist in delivery of health care in Italy.	Manfrin et al, 2017

SUPPLEMENTARY TABLE S4 – DETAILED CHARACTERISTICS OF INCLUDED ARTICLES AND STUDY-SITES

Abdel-Qader et al, 2022	*How many employees?*	Information not provided in the publication.
	What is the size of the pharmacy?	Information not provided in the publication.
	Where is it located related to other healthcare facilities?	A total of 49 community pharmacies in four different Jordanian regions were recruited to represent a cross-section of the community.
	How is a site different than others?	It was aimed to recruit community pharmacists with more than 6 years experience and those who were working in well-known pharmacies that are easily accessible to the public.
	What is the volume of prescriptions handled and how many customers?	Information not provided in the publication.
	Was any harm done?	No harm recorded in this study.
	How are pharmacists certified or were they?	Study pharmacists were provided with intensive training on research method and health coaching principles and techniques.<br><br>Training included discussions about health coaching, communication skills, and the concept of behavioural change process in light of self-determination theory, self-concordance theory, the transtheoretical model, and the COM-B model.
	What are the checks and balances?	A data reporting form was developed. Participants were followed-up two weeks post-intervention.
	How was inappropriate prescribing to sell more medications prevented or checked?	N/A
	Were there conflicts of interest?	No conflict of interest was declared in this study.
	What motivated the study?	As a result of the emerging necessities to enhance the role of the pharmacist and their awareness to assist patients in the management of COVID-19, a new concept was found to positively motivate patients in behaviours modification.
	What were the before and after in several areas?	Pharmacist-based virtual health coaching may be a potential strategy to advocate positive behaviours that can dampen the spread of COVID-19. This may possibly be applied to other disciplines.
Al Hamarneh et al, 2017	*How many employees?*	Information not provided in the publication.
	What is the size of the pharmacy?	Information not provided in the publication.
	Where is it located related to other healthcare facilities?	Information not provided in the publication.
	How is a site different than others?	Information not provided in the publication.

	What is the volume of prescriptions handled and how many customers?	Information not provided in the publication.
	Was any harm done?	No adverse events were self-reported during the study.
	How are pharmacists certified or were they?	The study was carried out in line with the study protocol and treatment recommendations, if made, were done so based on the most up-to-date Canadian clinical practice guidelines available at the time the study was conducted.
	What are the checks and balances?	The study team facilitated random checks of a sample of patients to monitor study sites' reported data and subsequently compare them with source documents so as to ensure reporting accuracy.
	How was inappropriate prescribing to sell more medications prevented or checked?	Treatment recommendations were made according to the most up-to-date Canadian clinical practice guidelines available at the time the study was conducted.
	Were there conflicts of interest?	No conflict of interest was identified.
	What motivated the study?	Pharmacists in Alberta can order and interpret laboratory tests, conduct medication management assessment and prescribe medications. This type of expanded scope of community pharmacy practice, in conjunction with the fact that around half of the community-dwelling patients with T2DM are not at their glycaemic targets, provide a unique opportunity for the use of community-pharmacy based interventions to supplement the identification and management of such high-risk patients.
	What were the before and after in several areas?	Community pharmacy-based case finding and intervention program reduced the risk for major CV events by 21% when compared to usual practice.
Cameron et al, 2020	*How many employees?*	Information not provided in the publication.
	What is the size of the pharmacy?	As per the paper, the study *"included a mix of large chain and small independent pharmacies that dispensed emergency contraception at a high volume, so the results are probably generalisable to UK pharmacies in which most emergency contraception is provided."*
	Where is it located related to other healthcare facilities?	Study pharmacies were chosen according to being within 5 miles of the sexual and reproductive health clinics.
	How is a site different than others?	Information not provided in the publication.
	What is the volume of prescriptions handled and how many customers?	Around more than 30 emergency contraception was dispensed per month.
	Was any harm done?	No identified harm.<br><br>There was a concern that a 3-month supply of the progestogen-only-pill would be too much and that motivation to seek subsequent effective contraception would fade, but findings from this study demonstrated otherwise.
	How are pharmacists certified or were they?	Training for pharmacists prior to delivering the intervention was provided using the study protocol and Patient Group Directions for the progestogen-only-pill.

	What are the checks and balances?	The proportion of women subsequently taking effective contraception as a result of the intervention was 20.1% greater than when compared to the control group. This difference was still significant after adjusting for co-factors, including age, current sexual relationship, history of effective contraception use, and also accounted for missing data (assuming missingness at random) – where the robustness of findings was still evident.<br><br>Mystery-shopper was used in this study.
	How was inappropriate prescribing to sell more medications prevented or checked?	In this trial, the intervention at hand was supply of the progestogen-only-pill. There was no upselling of other medication. To ensure that the progestogen-only-pill was appropriate to supply, women needed to be aged 16 years or older, not already using hormonal contraception, not on medication that could interfere with the progestogen-only-pill, and willing to provide contact details for follow-up.
	Were there conflicts of interest?	As per the Bridge-It study:<br><br>*"STC reports grants from the National Institute for Health Research (Health Technology Assessment [NIHR HTA] Programme), during the conduct of the study. AG is a consultant to HRA Pharma. AR reports receiving research grants from Gilead Sciences, Bristol-Myers Squibb, AbbVie, and Roche; honorariums from Gilead Sciences; and personal fees from AbbVie. LM and SP report funding from the UK Medical Research Council and Scottish Government Chief Scientist Office (Central Statistics Office) at the University of Glasgow (MC_UU_12017/11, SPHSU11). PB is a clinical director of the not-for profit community interest company SH:24, that provides online sexual health services in partnership with the UK National Health Service. KC reports being an employee of Boots UK, during the conduct of this study. AleM reports grants from NIHR HTA, during the conduct of this study. AleM is a clinical support bank midwife for SH:24 and a research midwife at Oxford University. JN was a deputy chair of the NIHR HTA General Board Committee (2016–19). All other authors declare no competing interests. This research is funded by the NIHR HTA project 15/113/01."*
	What motivated the study?	Women can access the emergency contraceptive pill through their local community pharmacy, in the UK. However, to receive ongoing supply of contraceptive medication after this point, patients would require a prescription from their GP. Pharmacists can only supply barrier methods, which have high failure rates. Securing a GP appointment can be difficult and time-ineffective to access. This in turn can increase the risk for unintended pregnancy.
	What were the before and after in several areas?	The proportion of women subsequently taking effective contraception as a result of the intervention was 20.1% greater than when compared to the control group.
Durrer et al, 2019	*How many employees?*	Information not provided in the publication.
	What is the size of the pharmacy?	Information not provided in the publication.
	Where is it located related to other healthcare facilities?	Information not provided in the publication.
	How is a site different than others?	Information not provided in the publication.
	What is the volume of prescriptions handled and how many customers?	Information not provided in the publication.

	Was any harm done?	There were four adverse events reported in the intervention group.<br><br>Three of the adverse events were related to mild hypoglycaemia.<br><br>The final adverse event was a cardiac event that occurred three weeks into the study and was deemed not related to the intervention.
	How are pharmacists certified or were they?	Certification not outlined in paper.
	What are the checks and balances?	Using a standardised medication description plan allowed for consistent implementation of the intervention in a safe and scalable manner.
	How was inappropriate prescribing to sell more medications prevented or checked?	Medication description plan.
	Were there conflicts of interest?	No conflict of interest declared in this study.
	What motivated the study?	Because of the need to reduce or eliminate glucose-lowering medications when T2DM patients follow a very low-carbohydrate or low-calorie diet, community pharmacists may be ideally positioned to safely and effectively deliver nutrition interventions and promoting T2DM remission.
	What were the before and after in several areas?	Intervention was effective in reducing the need for glucose-lowering medications and reduced medication effect score compared to treatment as usual.
Falamic et al, 2018	*How many employees?*	The name of these pharmacies and other demographic and logistical details are not outlined. It would require contacting the co-authors/Chief Investigators for additional information not available in this paper (for the questions identified).<br><br>The community pharmacy is situated in Donji Miholjac, Croatia. This particular community pharmacy is part of a primary care medical centre that consists of 9 general practitioner's ambulances, a laboratory, a paediatric and gynaecologist ambulance, radiology with X-ray and ultrasound, and four dentist ambulances.
	What is the size of the pharmacy?	
	Where is it located related to other healthcare facilities?	
	How is a site different than others?	
	What is the volume of prescriptions handled and how many customers?	
	Was any harm done?	None recorded

	How are pharmacists certified or were they?	As per protocol.
	What are the checks and balances?	Time in therapeutic range (TTR) was calculated by the Rosendaal method
	How was inappropriate prescribing to sell more medications prevented or checked?	Investigator education<br><br>Follow-up plan<br><br>Provided a pill-box for warfarin therapy only and participants were instructed to fill the pill-box according to the dosing scheme<br><br>Medication review was facilitated to avoid potential drug-interactions with warfarin<br><br>If necessary, the GP was contacted with recommendations for drug change or dose modification<br><br>Participants were instructed to let the research team know if they started a new drug, for the purpose of capturing potential drug-interactions
	Were there conflicts of interest?	None declared.
	What motivated the study?	There are currently no randomised control trials exploring the impact of community pharmacist's interventions on optimising warfarin treatment in the population of rural elderly patients.
	What were the before and after in several areas?	Number of INR values outside the therapeutic range were significantly higher in the control than in the intervention group
Heaton et al, 2018	*How many employees?*	Information not provided in the publication.
	What is the size of the pharmacy?	Information not provided in the publication.
	Where is it located related to other healthcare facilities?	Hybrid studies between six hospitals and a supermarket chain with 60 pharmacies.
	How is a site different than others?	Information not provided in the publication.
	What is the volume of prescriptions handled and how many customers?	Information not provided in the publication.
	Was any harm done?	No harm recorded in this study.
	How are pharmacists certified or were they?	All Kroger pharmacists previously received training in the provision of MTM services<br><br>Additional training was provided to around 200 Kroger pharmacists in the Cincinnati area to reinforce patient coaching skills, to identify unique aspects of care for patients transitioning from the hospital to home, and to review the specific disease states targeted in the project

	What are the checks and balances?	Descriptive statistics to measure differences in the independent variables between the intervention and control groups
	How was inappropriate prescribing to sell more medications prevented or checked?	Medication reconciliation between discharge plan and current therapy
	Were there conflicts of interest?	None declared
	What motivated the study?	Community pharmacists are in an ideal position to provide bridging-interventions, especially in the space of transition-of-care initiatives thereby reducing subsequent hospital readmission rates.
	What were the before and after in several areas?	Large-scale transition of care program between multiple health systems and community pharmacies are possible to reduce hospital readmissions. Community pharmacists are integral in this model of care.
Ibrahim et al, 2021	*How many employees?*	Information not provided in the publication.
	What is the size of the pharmacy?	Information not provided in the publication.
	Where is it located related to other healthcare facilities?	Information not provided in the publication.
	How is a site different than others?	Information not provided in the publication.
	What is the volume of prescriptions handled and how many customers?	Information not provided in the publication.
	Was any harm done?	No harm noted in this study.
	How are pharmacists certified or were they?	Study pharmacists underwent comprehensive online training on theoretical and practical aspects of health coaching delivered by the main author of this study.<br><br>Study pharmacists were also trained as to how to recruit and deal with participants.<br><br>The principle of behaviour change was explained to pharmacists using the COM-B model.
	What are the checks and balances?	To avoid bias, females who were exposed to breast-cancer awareness programmes or workshops were excluded from this study.
	How was inappropriate prescribing to sell more medications prevented or checked?	N/A
	Were there conflicts of interest?	No declarations of interest to declare.
	What motivated the study?	Need for a strategy that not only increases women's awareness of breast cancer, but also assists in the integration of behaviour change recommendations to their lives, health coaching is an innovative educational strategy that focuses not just on improving the outcomes of the disease, but also accounts for the patients' needs and feelings.

	What were the before and after in several areas?	Breast cancer-related health behaviours and knowledge can be improved through pharmacist-based health coaching.
Jahangard-Rafsanjani et al, 2018	*How many employees?*	Information not provided in the publication.
	What is the size of the pharmacy?	Information not provided in the publication.
	Where is it located related to other healthcare facilities?	This study was facilitated at the Ncmoonch-Talcghani Community Pharmacy, situated in Tehran. The community pharmacy is located in the northern part of Tehran where the socioeconomic status is relatively high. An endocrinologist whose office was located near the pharmacy was invited to collaborate with the research team and facilitate patients' recruitment.
	How is a site different than others?	Information not provided in the publication.
	What is the volume of prescriptions handled and how many customers?	Information not provided in the publication.
	Was any harm done?	None declared in this study.
	How are pharmacists certified or were they?	Community pharmacists were trained before the study commencement.<br><br>Pharmacists were trained by diabetes pharmacotherapy specialists in pathophysiology and pharmacotherapy of diabetes. After, pharmacists participated in a 3-day workshop for health care professionals on diabetes education.
	What are the checks and balances?	The community pharmacist used a predefined checklist to document the education procedure for each patient during the study period.<br><br>Participants were trained as to how to use their blood-glucose monitoring device.<br><br>Each patient was provided with a special logbook and educational pamphlets for the diabetes medications.<br><br>Participants were referred to a physician whenever the disease was not controlled after the first 2 months of intervention implementation or a drug therapy modification was required.
	How was inappropriate prescribing to sell more medications prevented or checked?	Participants were referred to a physician whenever the disease was not controlled after the first 2 months of intervention implementation or a drug therapy modification was required.
	Were there conflicts of interest?	None declared in this study.
	What motivated the study?	Community pharmacists have expanded their professional roles beyond dispensing medications, especially in the space of diabetes screening and management. It is recognised that registered pharmacists are vital members of the multidisciplinary teams responsible for delivering diabetes care and education.
	What were the before and after in several areas?	Further studies are required to quantify the effectiveness of blood-glucose monitoring devices in the clinical settings of resource-limited countries.

Kooij et al, 2016	*How many employees?*	Information not provided in the publication.
	What is the size of the pharmacy?	Information not provided in the publication.
	Where is it located related to other healthcare facilities?	Information not provided in the publication.
	How is a site different than others?	Information not provided in the publication.
	What is the volume of prescriptions handled and how many customers?	Information not provided in the publication.
	Was any harm done?	None declared in this study.
	How are pharmacists certified or were they?	Pharmacists received a three-hour training aimed at understanding beliefs and behavior of patients related to medication intake.<br><br>Training included case-studies and an assessment of the level of theoretical knowledge on communication.
	What are the checks and balances?	Patients in the intervention arm were selected weekly through an automated selection procedure.
	How was inappropriate prescribing to sell more medications prevented or checked?	The purpose of the tele-intervention was to improve medication adherence. This was compartmentalised as acquiring the need for information; actual medication intake behaviour; practical barriers including side-effects; perceptual barriers including concerns or low necessity beliefs.
	Were there conflicts of interest?	None declared in this study.
	What motivated the study?	Telephone counselling may be a feasible alternative to face-to-face counselling:<br>- Patient may feel more comfortable when approached in their own environment where lack of privacy is not an issue.<br>- Patients who are not able to visit a pharmacy can be reached.<br>Pharmacists can prepare themselves on the call and the telephone calls can be planned.
	What were the before and after in several areas?	Patients initiating antidepressants did not benefit from the intervention.
Krass et al, 2017	*How many employees?*	Information not provided in the publication.
	What is the size of the pharmacy?	Information not provided in the publication.
	Where is it located related to other healthcare facilities?	96 pharmacies (metropolitan)<br><br>80 (regional)<br><br>24 (remote)

		A geographical sampling method for enrolment was used to acknowledge access and socio-economic conditions at the postcode level. This method was applied systematically to each Australian State and Territory.
	How is a site different than others?	Information not provided in the publication.
	What is the volume of prescriptions handled and how many customers?	Information not provided in the publication.
	Was any harm done?	No harm was identified.
	How are pharmacists certified or were they?	In order to be able to commence screening, participating pharmacists were required to complete the Continuing Professional Development accredited online training course and assessment to highlight competence in using the relevant POC device.
	What are the checks and balances?	Pre-specified criteria for referral confirmed diagnosis of diabetes in patients by GPs once a full assessment of the patient was carried out.
	How was inappropriate prescribing to sell more medications prevented or checked?	N/A
	Were there conflicts of interest?	No conflict of interest.
	What motivated the study?	A lack of head-to-head trials comparing different approaches means that it is unknown as to the most clinically effective and cost-effective screening model for community pharmacy.
	What were the before and after in several areas?	At current, the standalone AUSDRISK risk calculation tool is the standard of care. Although implementing the POC tests in conjunction with the AUSDRISK, was more effective in identifying new cases of T2DM in a community pharmacy setting compared to risk assessment only.
Manfrin et al, 2017	*How many employees?*	Information not provided in the publication.
	What is the size of the pharmacy?	Information not provided in the publication.
	Where is it located related to other healthcare facilities?	Information not provided in the publication.
	How is a site different than others?	Information not provided in the publication.
	What is the volume of prescriptions handled and how many customers?	Information not provided in the publication.
	Was any harm done?	No harm was identified in this study.
	How are pharmacists certified or were they?	Community pharmacists were trained to identify pharmaceutical care issues that may impact on optimal medicine use or asthma control, and provide advice to the patients and recommendations to their GPs, as required.
	What are the checks and balances?	Per protocol analysis.

	How was inappropriate prescribing to sell more medications prevented or checked?	N/A
	Were there conflicts of interest?	No competing interests were declared.
	What motivated the study?	In Italy, Medicines Use Review has not been introduced. In this case, no empirical research exists to address the effectiveness of an MUR service in Italy. The MUR is a leading pharmacy-led approach in the management of asthma internationally.
	What were the before and after in several areas?	Prior to this trial, an MUR service was not established in Italy. This community pharmacy-based intervention that has demonstrated a high effectiveness in the management of asthma has since been implemented as the first community pharmacy cognitive service in Italy.<br><br>Patients receiving the intervention were 1.8 times more likely to improve from not controlled to controlled than control patients.
Paravattil et al, 2021	*How many employees?*	Information not provided in the publication.
	What is the size of the pharmacy?	Information not provided in the publication.
	Where is it located related to other healthcare facilities?	Six community pharmacies were pre-selected to obtain a representative and diverse sample of residents living in Qatar.
	How is a site different than others?	Information not provided in the publication.
	What is the volume of prescriptions handled and how many customers?	Information not provided in the publication.
	Was any harm done?	No harm identified in this study.
	How are pharmacists certified or were they?	Standardised instructions and training were provided to the pharmacists based on their group assignment.<br><br>Study pharmacists assigned to the structured counselling group received training on how to conduct a comprehensive medication review and antibiotic counselling to patients. Role plays and patient cases were used to supplement this.<br><br>Counselling for antibiotics followed the structure of: medication name, indication, dose, frequency, duration, side effects, precautions, administration, and how to handle missed doses.<br><br>A booklet of all available oral antibiotics in Qatar was provided for each study pharmacist in the counselling-arm of this study to use as a reference when counselling patients.

		Study pharmacists allocated to the call-back group received the same training as with the counselling-arm, with an additional one-hour training session on how to facilitate a call-back service. This included interventional approaches on how to manage antibiotic nonadherence, side effects, and ineffective therapy.
	What are the checks and balances?	All patients enrolled in the study were followed up by the research team within 1-2 days after completion of their antibiotic therapy to evaluate antibiotic adherence, symptom severity score, and satisfaction with the counselling provided by the study pharmacist.
	How was inappropriate prescribing to sell more medications prevented or checked?	The appropriateness of the prescribed antibiotic was evaluated by an Infectious Diseases' Expert.<br><br>Prescriptions were classified as appropriate based on the Sanford Guide to Antimicrobial Therapy 2020, antimicrobial prescribing policy of Hamad Medical Corporation, symptom presentation, and clinical judgement.
	Were there conflicts of interest?	The authors declare no conflict of interest.
	What motivated the study?	Misuse of antibiotics, inappropriate prescribing of antibiotics, community pharmacists are heavily involved in medication-centred roles such as dispensing with limited opportunity to perform patient-centred care services.
	What were the before and after in several areas?	The call-back and structured counselling study arms promoted high antibiotic adherence rates when compared to the standard care group.
Tsuyuki et al, 2015<br><br>R$_z$ACTION	*How many employees?*	Information not provided in the publication.
	What is the size of the pharmacy?	Information not provided in the publication.
	Where is it located related to other healthcare facilities?	Information not provided in the publication.
	How is a site different than others?	Information not provided in the publication.
	What is the volume of prescriptions handled and how many customers?	Information not provided in the publication.
	Was any harm done?	No adverse event recorded in this trial.
	How are pharmacists certified or were they?	Pharmacists education was provided prior to implementation of intervention. Pharmacists in this study had already been awarded with a prescribing authority.
	What are the checks and balances?	CHEP guidelines<br><br>Patient's primary care physician was notified of all assessment results and drug therapy changes in person or by fax.
	How was inappropriate prescribing to sell more medications prevented or checked?	Patient's primary care physician was notified of all assessment results and drug therapy changes in person or by fax.
	Were there conflicts of interest?	Dr Tsuyuki has received research funds for investigator-initiated trials from AstraZeneca, Sanofi, and Merck and has provided consulting for PharmaSmart International and Boehringer Ingelheim. The other authors report no conflicts.

	What motivated the study?	Allowing community pharmacists to independently prescribe drug therapy may result in even better patient outcomes than interventions based solely on providing recommendations.
	What were the before and after in several areas?	Pharmacist prescribing for patients with hypertension yielded a clinically important and statistically significant reduction in elevated blood-pressure.
Tsuyuki et al, 2015<br><br>R_zACTION	*How many employees?*	Information not provided in the publication.
	What is the size of the pharmacy?	Information not provided in the publication.
	Where is it located related to other healthcare facilities?	Information not provided in the publication.
	How is a site different than others?	Information not provided in the publication.
	What is the volume of prescriptions handled and how many customers?	Information not provided in the publication.
	Was any harm done?	No adverse event recorded in this trial.
	How are pharmacists certified or were they?	Pharmacists education was provided prior to implementation of intervention. Pharmacists in this study had already been awarded with a prescribing authority.
	What are the checks and balances?	CHEP guidelines<br><br>Patient's primary care physician was notified of all assessment results and drug therapy changes in person or by fax.
	How was inappropriate prescribing to sell more medications prevented or checked?	Patient's primary care physician was notified of all assessment results and drug therapy changes in person or by fax.
	Were there conflicts of interest?	Dr Tsuyuki has received research funds for investigator-initiated trials from AstraZeneca, Sanofi, and Merck and has provided consulting for PharmaSmart International and Boehringer Ingelheim. The other authors report no conflicts.
	What motivated the study?	Allowing community pharmacists to independently prescribe drug therapy may result in even better patient outcomes than interventions based solely on providing recommendations.
	What were the before and after in several areas?	Pharmacist prescribing for patients with hypertension yielded a clinically important and statistically significant reduction in elevated blood-pressure.

Supplementary Figure S1 – PRISMA Flow Chart

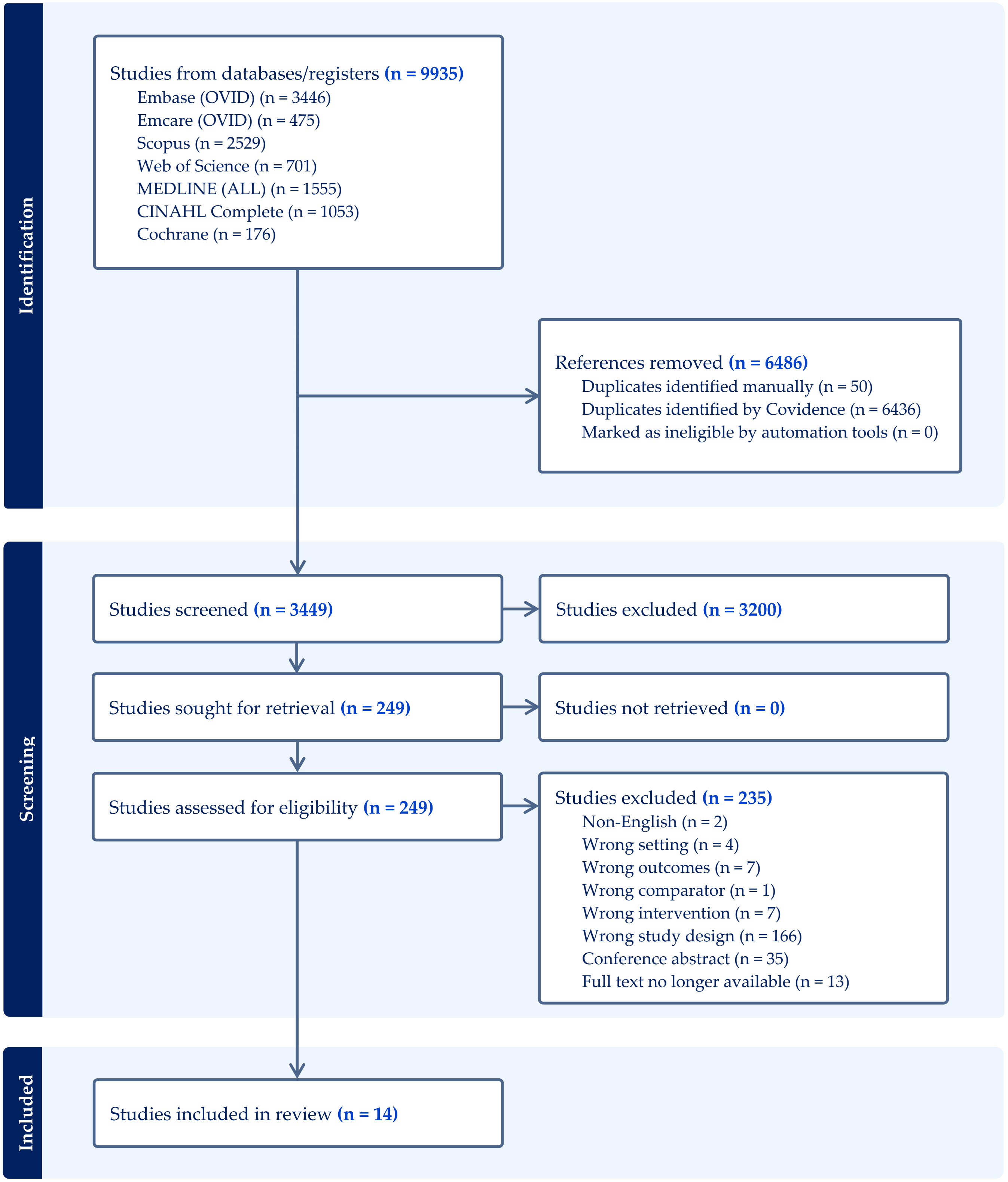

Supplement: Supplementary file 1 [file pharmacy-12-00095-s001.zip › pharmacy-3013217-supplementary.pdf]
